# Progesterone receptor potentiates macropinocytosis through CDC42 in pancreatic ductal adenocarcinoma

**DOI:** 10.1038/s41389-024-00512-7

**Published:** 2024-02-29

**Authors:** Ying-Na Liao, Yan-Zhi Gai, Li-Heng Qian, Hong Pan, Yi-Fan Zhang, Pin Li, Ying Guo, Shu-Xin Li, Hui-Zhen Nie

**Affiliations:** 1 State Key Laboratory of Systems Medicine for Cancer, Shanghai Cancer Institute, Ren Ji Hospital, School of Medicine, Shanghai Jiao Tong University, Shanghai, 200240 P.R. China; 2 The International Peace Maternity and Child Health Hospital, School of Medicine, Shanghai Jiao Tong University, Shanghai, 20030 P.R. China; 3 Radiology Department, The First Affiliated Hospital, Zhejiang University School of Medicine, Hangzhou, 310000 P.R. China

**Keywords:** Pancreatic cancer, Cancer metabolism

## Abstract

Endocrine receptors play an essential role in tumor metabolic reprogramming and represent a promising therapeutic avenue in pancreatic ductal adenocarcinoma (PDAC). PDAC is characterized by a nutrient-deprived microenvironment. To meet their ascendant energy demands, cancer cells can internalize extracellular proteins via macropinocytosis. However, the roles of endocrine receptors in macropinocytosis are not clear. In this study, we found that progesterone receptor (PGR), a steroid-responsive nuclear receptor, is highly expressed in PDAC tissues obtained from both patients and transgenic *LSL-Kras*^*G12D/+*^*; LSL-Trp53*^*R172H/+*^*; PDX1-cre* (KPC) mice. Moreover, PGR knockdown restrained PDAC cell survival and tumor growth both in vitro and in vivo. Genetic and pharmacological PGR inhibition resulted in a marked attenuation of macropinocytosis in PDAC cells and subcutaneous tumor models, indicating the involvement of this receptor in macropinocytosis regulation. Mechanistically, PGR upregulated CDC42, a critical regulator in macropinocytosis, through PGR-mediated transcriptional activation. These data deepen the understanding of how the endocrine system influences tumor progression via a non-classical pathway and provide a novel therapeutic option for patients with PDAC.

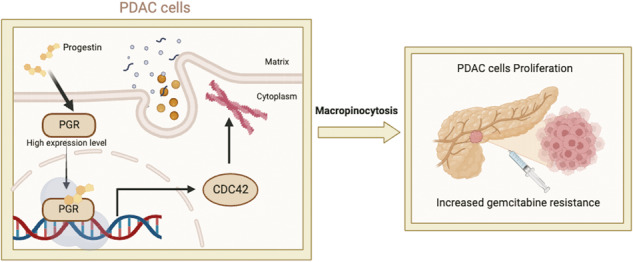

## Introduction

Pancreatic ductal adenocarcinoma (PDAC) is a highly lethal disease characterized by limited therapeutic options and a notoriously dismal prognosis. Despite the recent 5-year survival rate being 8%, progress in therapies remains sluggish [1]. PDAC is initiated by KRAS mutations and subsequently several genetic alterations such as CDKN2A, TP53, and SMAD4, leading to the progression of pancreatic intraepithelial neoplasms (PanIN) to invasive and metastatic PDAC [2]. Moreover, the PDAC tumor microenvironment features abundant stromal cells with excessive extracellular matrix (ECM), resulting in low nutrient availability, poor oxygen diffusion, and high pressure [3, 4].

Macropinocytosis is an actin-dependent endocytic mechanism that enables the “drinking” of extracellular fluids containing a diverse range of proteins and macromolecules within endocytic vacuoles (> 0.2 μm) [5]. This process provides an essential role in various physiological activities. For instance, dendritic cells and macrophages employ macropinocytosis to surveil antigens/microbial-associated molecules, whereas rapidly proliferating cancer cells acquire nutrients robustly to fuel their growth [6–9]. Tumors driven by KRAS, such as PDAC, are characterized by heightened levels of macropinocytosis [10, 11]. In PDAC tumor cells, macropinocytosis is used to scavenge and catabolize extracellular protein to circumvent the stresses of a nutrient-deprived milieu [12]. The scarcity of amino acids in the tumor microenvironment could induce EGFR phosphorylation and subsequently up-regulate macropinocytosis via Pak activation [13]. Earlier studies have reported that RAS-mutant tumor cells use macropinocytosis to uptake and degrade proteins for entry into the central carbon pathway, yielding amino acids, by activating small GTPases such as Rac1 [14, 15].

Endocrine events play a vital role in metabolic processes, internal homeostasis, growth, and several pathologic alterations. Nuclear hormone receptors, such as estrogen receptors (ER), mineralocorticoid receptor (MR), and progesterone receptor (PGR), have the capacity to translate hormone signals into specific gene programs. Along this line, our team has previously documented the non-classical function of classical hormone receptors in tumor cell metabolism [16, 17]. As a member of the NRs, the progesterone receptor (PGR) targeted by progesterone is the key steroid receptor that regulates proliferation and differentiation in the mammary gland and reproductive tract [18]. Recent investigations have demonstrated that PGR can affect cancer-related processes during initiation and therapy; however, whether and how PGR contributes to pancreatic tumor cell metabolic reprogramming has not been addressed. In this study, we characterized the impact of PGR in upregulating macropinocytosis function via CDC42 in PDAC cells and promoting proliferation.

## Results

### PGR is a potential macropinocytosis regulator overexpressed in PDAC

**Fig. 1:**
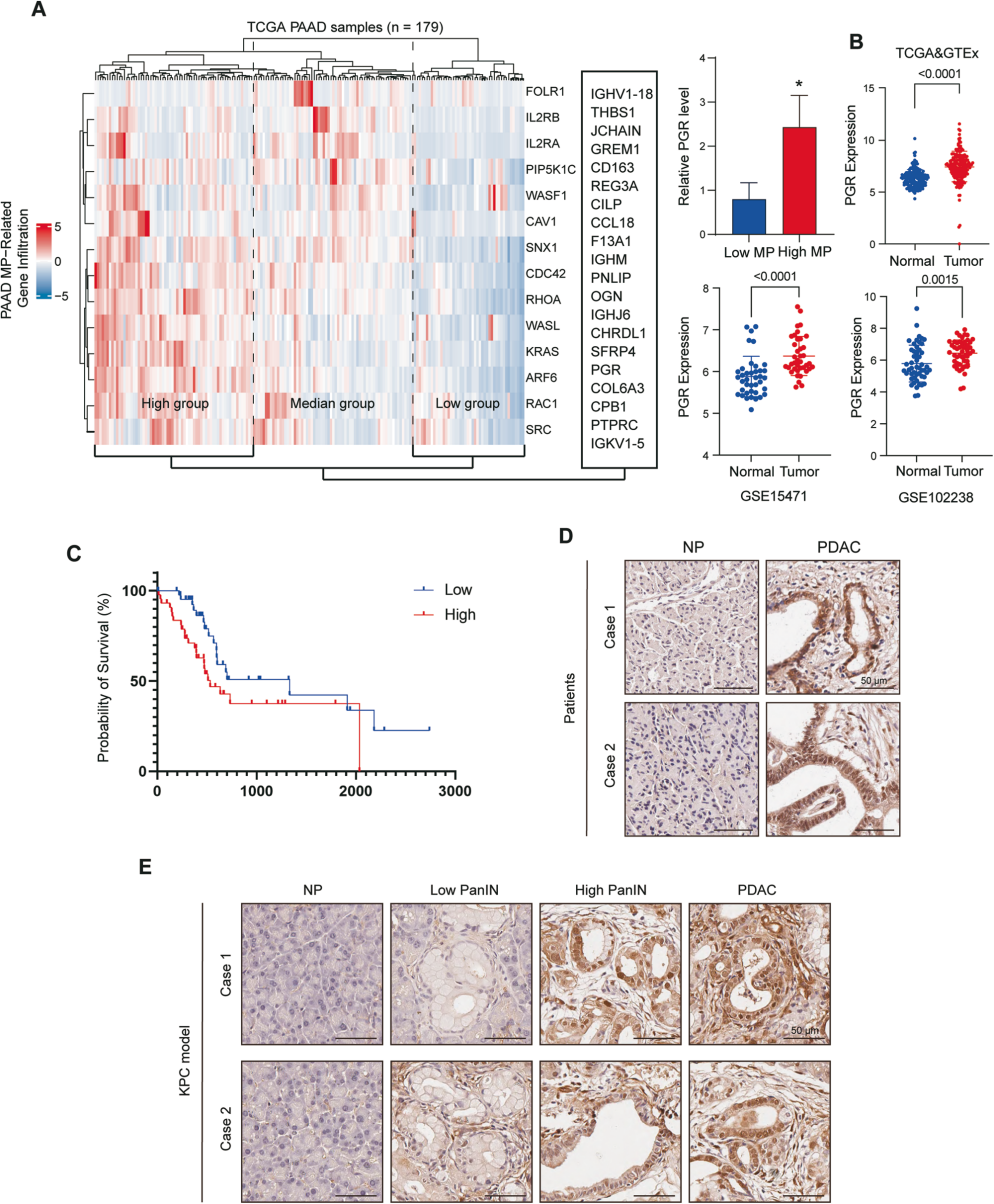
PGR is a potential macropinocytosis gene and is overexpressed in PDAC. **A** Hierarchical cluster analysis of genes related to macropinocytosis and the top 20 differently expressed genes between high and low macropinocytosis groups. Expression of PGR between high and low macropinocytosis groups (right panel). **B** Expression of PGR of paired normal pancreatic (NP) and pancreatic ductal adenocarcinoma (PDAC) tissues in TCGA&GTEx, GSE15471, GSE102238. *p* values were determined by paired two-tailed *t*-test. **C** Kaplan–Meier curves of overall survival of pancreatic patients stratified by PGR expression. Data were obtained from the TCGA. *n* = 91 patients. **D** Representative images of immunohistochemical staining for PGR in pancreatic cancer tissues and normal pancreatic tissues from two cases of patients. Scale bar, 50 μm. **E** Representative images of immunohistochemical staining for PGR in pancreatic tissues from KPC mice. Scale bar, 50 μm.

To investigate the role of macropinocytosis in PDAC samples, we conducted Gene set variation analysis (GSVA) to examine the differentially expressed macropinocytosis signatures in RNA-Seq data of PAAD patients from The Cancer Genome Atlas (TCGA) database. Utilizing the Single-sample Gene Set Enrichment Analysis (ssGSEA) score, we performed unsupervised hierarchical cluster analysis to group the samples into three subgroups: “High-macropinocytosis”, “Median-macropinocytosis”, and “Low-macropinocytosis”. We then identified the top 20 differentially expressed genes (DEGs) between high- and low-macropinocytosis groups and found that PGR, a nuclear hormone receptor, was upregulated in the high-macropinocytosis group. This observation suggested that PGR could be a potential gene of interest for promoting pancreatic tumor progression via macropinocytosis (Fig. 1A).

**Fig. 2:**
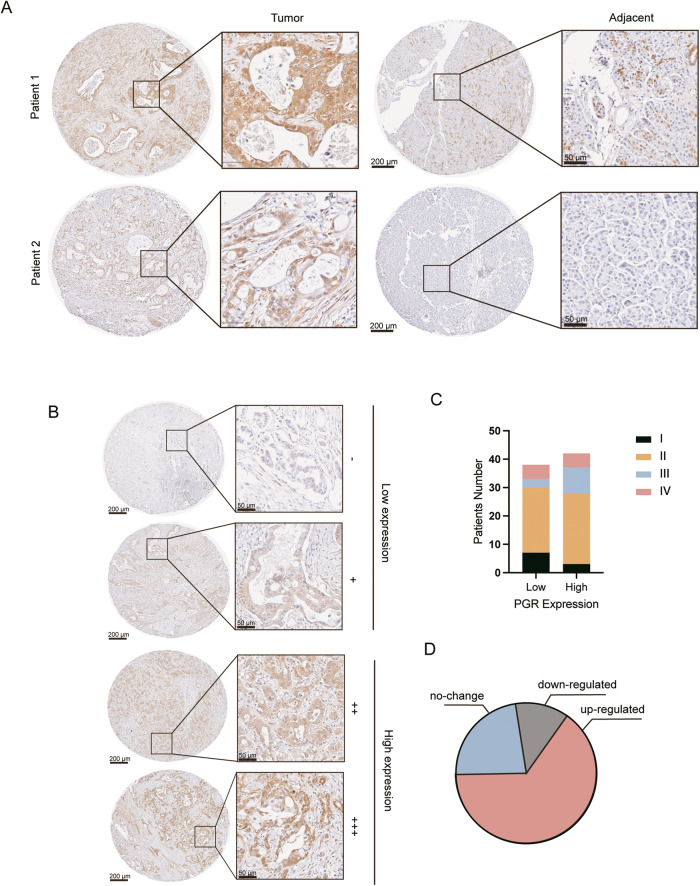
PGR is overexpressed in PDAC patients and related to the clinicopathologic parameters. **A** Representative images of immunohistochemical staining for PGR in pancreatic tissues tissue microarray. Scale bar, 200 μm (left panel) and 50 μm (right panel). **B**–**D** Representative IHC staining (**B**) and statistical analysis of the PGR levels with pancreatic cancer TNM stages (**C**). Scores −, + represent low expression and ++, +++ represent high expression. **D** Quantification of PGR up-regulated, down-regulated, and no change cases of PDAC pancreatic tissue microarray. (*n* = 80).

We further examined PGR expression patterns in the GEO data sets (GSE15471, GSE102238), and found that PGR expression was increased in PDAC compared to normal pancreas tissue. We also analyzed combined TCGA and GTEx data, which confirmed our findings (Fig. 1B). Survival analysis indicated that higher expression of PGR correlated with worse survival in PDAC patients (Fig. 1C). The immunohistochemical staining of a tissue panel of the normal pancreas (NP) and PDAC showed heightened PGR expression in PDAC relative to NP (Fig. 1D). The pancreatic tissues in KPC mice exhibit precursor lesions of pancreatic tumors, pancreatic intraepithelial neoplasias (PanIN), and locally invasive pancreatic ductal adenocarcinoma (PDAC), accompanied by extensive stromal fibrosis, closely resembling the morphological observations in human pancreatic cancer tissues. To explore whether PGR is involved in PDAC progression, we performed immunohistochemical (IHC) analysis on pancreatic tissues derived from KPC mice. We found that PGR expression was increased in mouse PanIN cells and PDAC cells compared with adjacent normal cells, suggesting that elevated PGR expression may be associated with PDAC tumor initiation (Fig. 1E). IHC analysis on the pancreatic tissue microarrays (TMAs) from 80 patients confirmed that PGR was up-regulated in 64.9% of PDAC tissues, with a positive association between higher PGR expression and TNM stage (Fig. 2A–D). These data suggested that PGR was overexpressed in pancreatic cancer and related to the progression of PDAC.

### PGR contributes to cell survival and tumor growth in PDAC

PGR is expressed in a wide variety of human tissues, existing as two major isoforms, PGR-A and PGR-B. The previous study using the PGR-specific antibody immunolocalized the expression of different PGR isoforms in various human tissues and showed that the expression patterns of PRA and PRB have organ-specificity. Although PGR-A and PGR-B were detected in the nucleus and cytoplasm of pancreatic acinar cells, relatively high levels of PGR-B were reported compared to the PRA [19].

**Fig. 3:**
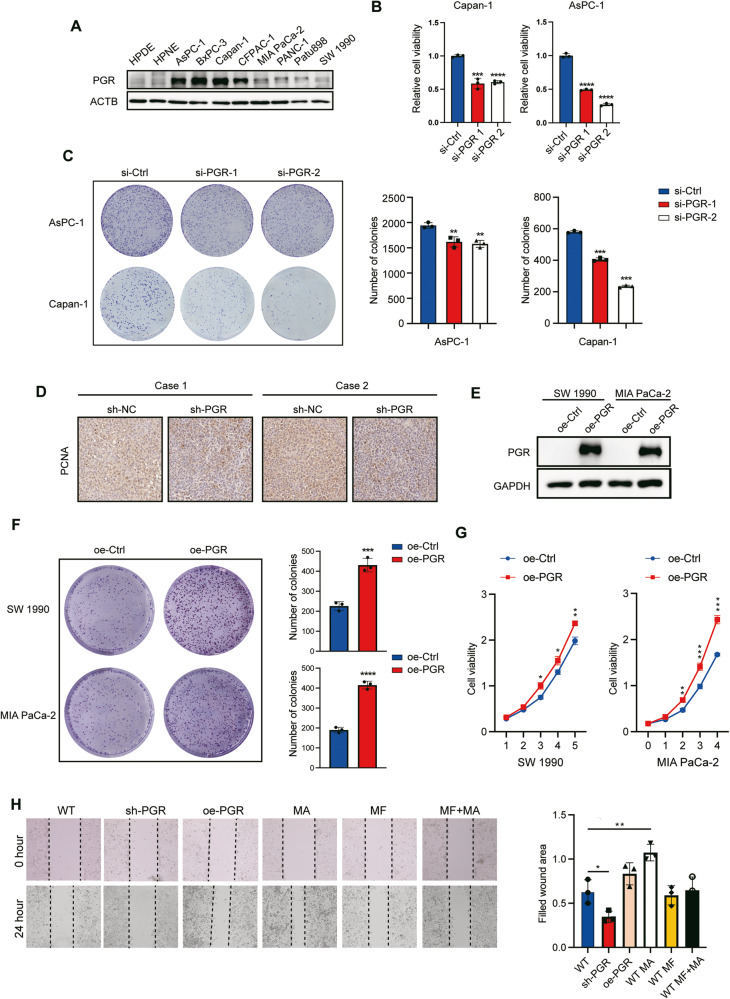
PGR contributes to cell survival and tumor growth in PDAC. **A** Western blot analysis of PGR expression levels in human PDAC cell lines. **B** CCK-8 assay of PGR knockdown by siRNA in Capan-1 cells (left) and AsPC-1 cells (right) (*n* = 5). Statistical significance was determined using *t*-test. *p ≤ 0.05, **p ≤ 0.01, ***p ≤ 0.001. **C** Colony formation assay of control AsPC-1 vs AsPC-1 with PGR knockdown (top) and control Capan-1 vs Capan-1 with PGR knockdown (bottom) (*n* = 3). The right panel is quantification. Statistical significance was determined using *t*-test. *p ≤ 0.05, **p ≤ 0.01, ***p ≤ 0.001. **D** PCNA immunostaining in xenograft pancreatic tumor sections in control and PGR knockdown group. Scale bar, 100 μm. **E** Western blot analysis of PGR overexpression levels in SW1990 and MIA PaCa-2 cells. **F** Colony formation assay of cells overexpressed PGR and the quantification. **G** CCK-8 assay of PDAC cells overexpressed PGR. *p ≤ 0.05, **p ≤ 0.01, ***p ≤ 0.001, ****p ≤ 0.0001. **H** Wound healing assay of Capan-1 cell transfected with shRNA and overexpressed-plasmid or treated with Medroxyprogesterone acetate (MA) (10 μM) and Mifepristone (MF) (10 μM) for 24 h. *p ≤ 0.05, **p ≤ 0.01.

Through expression level detection, we identified several PDAC cell lines with high PGR expression levels (Fig. 3A). Among them, AsPC-1, BxPC-3, and Patu8988 were derived from female patients, while Capan-1 and CFPAC-1 originated from male patients. Therefore, it can be inferred that there is no clear correlation between PGR expression level and gender. We conducted functional studies using PDAC cell lines and observed a significant decrease in cell viability and colony formation in the PGR knockdown group (Fig. 3B, C), whereas PGR overexpression improved cell proliferation and colony formation in PDAC cells (Fig. 3E–G). To further validate the effect of PGR in mediating tumor growth in vivo, we developed a mice xenograft tumor model, implanting Capan-1 cells expressing control or shRNAs targeting PGR. The PCNA immunostaining revealed that Capan-1 cells bearing PGR shRNA showed inhibition of PDAC tumor progression (Fig. 3D). These results are consistent with our in vitro findings, indicating that PGR is essential for the tumorigenic abilities of PDAC cells. The wound healing assay showed that both overexpressing and activating PGR by pharmacologic agonist medroxyprogesterone acetate (MA) and antagonist Mifepristone (MF) were able to reinforce PDAC cell migration ability (Fig. 3H).

### PGR knockdown has a synergic effect with gemcitabine in PDAC cells

**Fig. 4:**
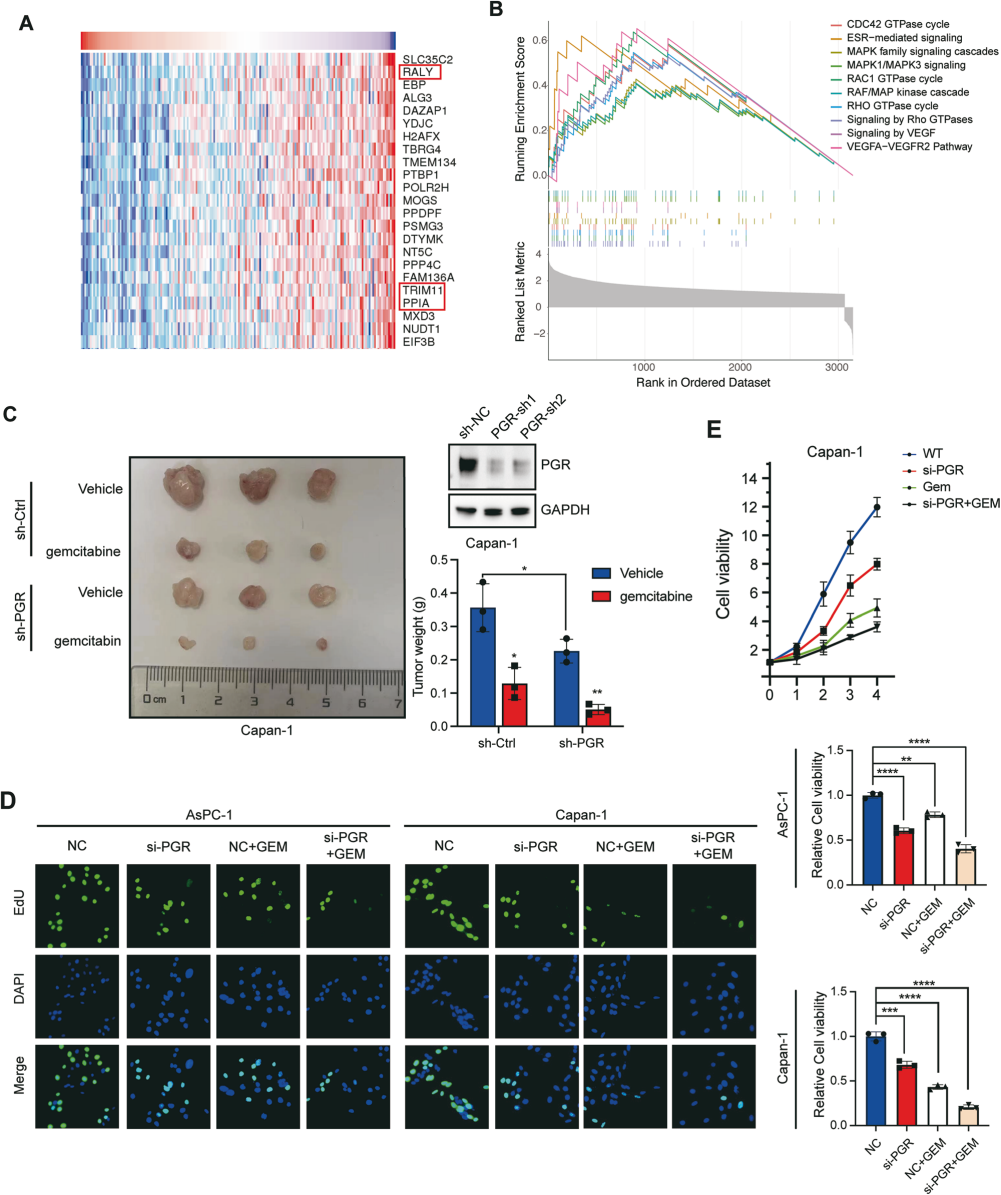
PGR knockdown has a synergic effect with gemcitabine in PDAC cells. **A** The co-expression analysis of PGR with PDAC RNA-sequence data in linkedomics dataset. **B** Reactome gene set enrichment analysis of PGR expressed-relative DEGs in TCGA database. The top 10 terms. **C** Subcutaneous xenografts of Capan-1 cells transfected with sh-Ctrl or sh-PGR and treated with or without gemcitabine (*n* = 3). Tumor weight was measured and compared between the four groups. Statistical significance was determined using unpaired t-test. **p ≤ 0.01. **D** EdU incorporation assay in PDAC cells (AsPC-1 and Capan-1) transfected si-NC or si-PGR and treated with gemcitabine. Scale bar, 50 μm. **E** CCK8 assay of PDAC cells (Capan-1) transfected si-NC or si-PGR and treated with gemcitabine. *p ≤ 0.05, **p ≤ 0.01, ***p ≤ 0.001.

Several studies revealed that PGR plays a noteworthy role in chemoresistance of endometrial tumors and breast tumors [20, 21]. We analyze the PGR expression-correlated genes by using the Linkedomics database (Fig. 4A). The results showed that the expression of RALY, a significantly negative factor in oxaliplatin resistance [22], was increased while PGR was downregulated. In addition, we queried the PDAC dataset from The Cancer Genome Atlas (TCGA) database of genomic expression information on 178 patients, and patients were subsequently sub-grouped into high-expressed and low-expressed based on their relative expression of PGR. To gain insights into the molecular landscape, we ranked the different expression genes between the PGR-high group and PGR-low group and subjected them to Reactome gene-set enrichment analyses (GSEA). The PGR-high tumor samples were enriched in the expression of gene signatures related to MAPK signaling and vascular endothelial growth factor pathway (Fig. 4B). Prior research showed that VEGF/VEGFR signaling in non-small-cell lung carcinomas was able to activate survival pathways and promote chemotherapeutic resistance [23, 24]. As reported, MAPKs were aberrantly activated in multiple cancers when acquired drug resistance [25, 26].

To further explore whether PGR contributes to the effectivity of gemcitabine (Gem) treatment, a first-line chemotherapy in PDAC, we developed a xenograft model, employing Capan-1 cells expressing control or sh-PGR, and treating with or without Gem. Notably, the sh-PGR group treated with GEM exhibited lower tumor weight compared to the Ctrl group (Fig. 4C). In addition, the suppression effect of Gem treatment on PDAC cell proliferation was enhanced by PGR knockdown (Fig. 4D, E). These findings suggest that the knockdown of PGR leads to a synergistic effect with gemcitabine treatment.

### PGR activates macropinocytosis-related pathway in PDAC cells

**Fig. 5:**
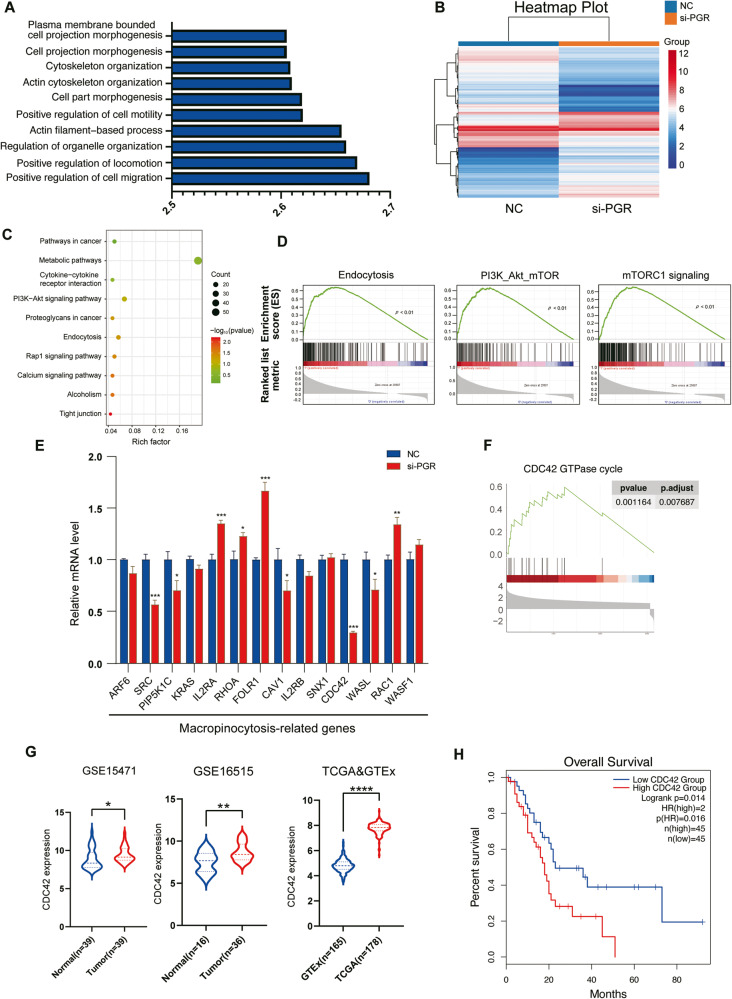
PGR activates macropinocytosis-relative pathways in PDAC cells. **A** GSEA analysis plot of GO-BP function in TCGA database. The top 15 terms. **B** Heatmap profiles of differential gene expression between negative control (NC) and si-PGR AsPC-1 cells from mRNA array analysis. Red indicates an increase in expression and blue indicates a decrease in expression. **C** KEGG pathways ranked by *p* value are significantly upregulated in PGR high-expression group. **D** Gene Set Enrichment Analysis (GSEA) of pathways related to cancer, mTORC1, PI3K-Akt-mTOR and endocytosis in mRNA array analysis data. **E** Expression changes of genes related to macropinocytosis between NC and si-PGR AsPC-1 cells. *t*-test was used to calculate the significance between NC and si-PGR groups. *p ≤ 0.05, **p ≤ 0.01, ***p ≤ 0.001. **F** Reactome Gene Set Enrichment Analysis related to CDC42GTPase cycle in TCGA dataset. **G** Expression of CDC42 of normal pancreatic (NP) and pancreatic ductal adenocarcinoma (PDAC) tissues in TCGA&GTEx, GSE15471, and GSE102238. *p* values were determined by paired two-tailed *t*-test. *p ≤ 0.05, **p ≤ 0.01, ***p ≤ 0.001, ****p ≤ 0.0001. **H** Kaplan–Meier curves of overall survival of pancreatic patients stratified by CDC42 expression. *n* = 90.

For GO enrichment analysis, the PGR-high tumor samples in TCGA database were enriched in the expression of gene signatures related to cytoskeleton organization, and cell migration, among others (Fig. 5A). To pinpoint the mechanism by which PGR affects PDAC cell behaviors, we further performed mRNA array analysis in control and PGR-knockdown PDAC cells. There were 858 and 647 genes up- and downregulated (Fig. 5B). KEGG pathway analysis of the differentially expressed genes showed significant alterations in the endocytosis pathway (Fig. 5C). Moreover, Gene set enrichment analysis (GSEA) shed light on the downregulation of macropinocytosis-related gene sets, such as endocytosis, PI3K-AKT-mTOR signaling, and mTORC1 signaling (Fig. 5D). In accordance with these findings, several genes concerned with macropinocytosis were altered (Fig. 5E).

Macropinocytosis is a specialized form of endocytosis that enables tumor cells to efficiently catabolize extracellular proteins under nutrient-poor conditions via large endocytic vacuoles. Enrichment analyses were also performed based on the DEGs described previously, GO analysis in the biological process suggested the significantly altered aspects were cell morphogenesis and cytoskeleton organization (Fig. 5A). The Reactome pathway analysis indicated several pathways associated with macropinocytosis were considerably changed such as “RHO GTPase cycle”, “RAC1 GTPase cycle”, and “CDC42 GTPase cycle” (Fig. 4B). Collectively, we hypothesized that PGR may regulate macropinocytosis in PDAC cells.

### PGR regulates macropinocytosis in PDAC cells in vitro and in vivo

**Fig. 6:**
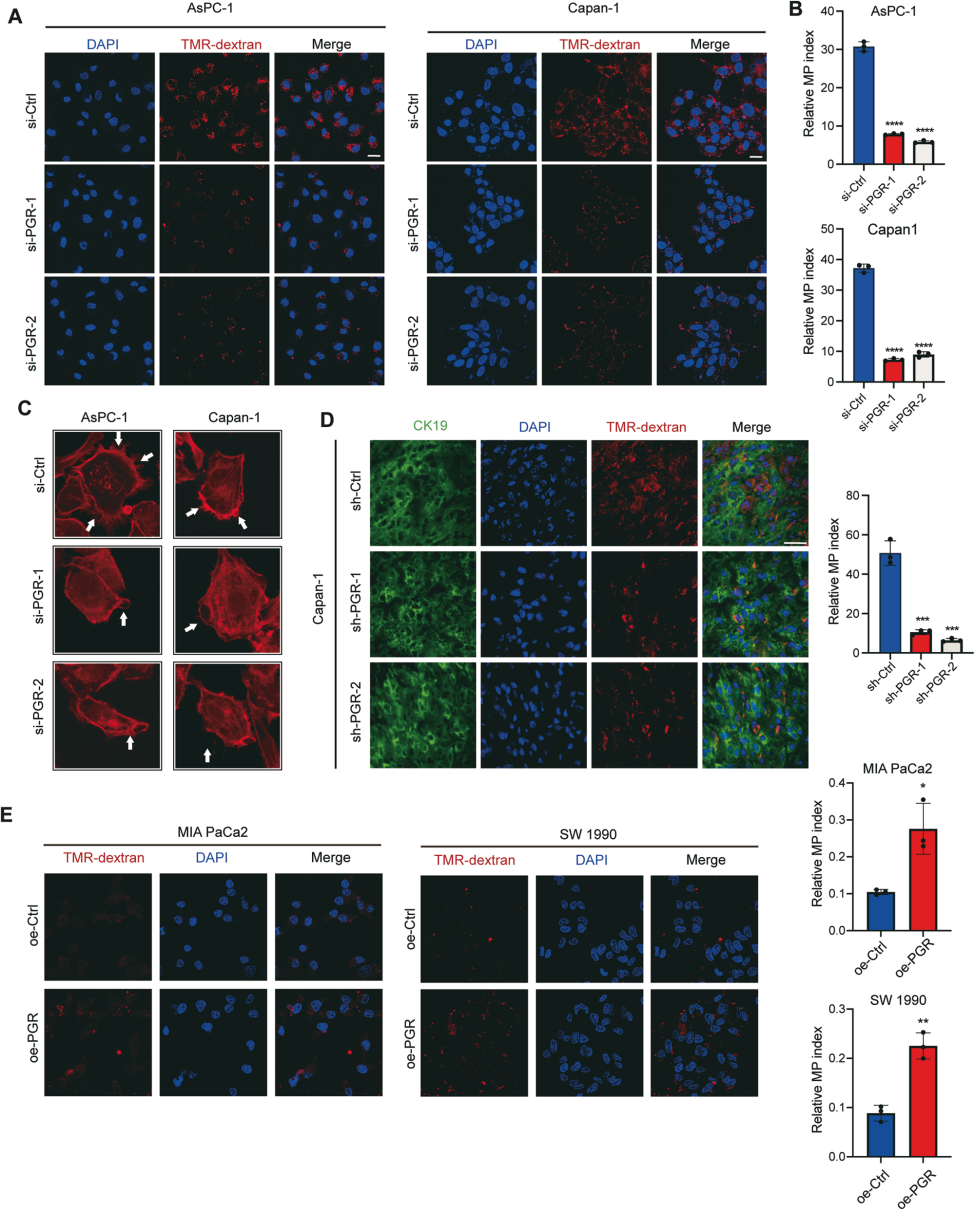
PGR regulates macropinocytosis in PDAC cells in vitro and in vivo. **A** Representative fluorescence images of dextran uptake by PDAC cells transfected with si-Ctrl or si-PGR. (Scale bar, 50 μm). **B** Quantification of macropinocytosis in **A**. *n* = 6 and *p*-value determined by *t*-test. **C** Representative images of Capan-1 and AsPC-1 cells transfected with si-Ctrl or si-PGR, with actin filaments labeled with phalloidin-iFluor 594 (red). White arrowheads indicate membrane ruffles. Scale bar, 10 μm. **D** Representative fluorescence images of dextran (red) uptake from sections of PDAC xenograft tumor tissue. Tumor cells immunostained with anti-CK19 (green) and nuclei are labeled with DAPI (blue). Scale bar, 50 μm. Quantification of macropinocytosis in PDAC xenograft tumor tissue (right panel). *n* = 5, and *p*-value is determined by *t*-test. **E** Representative fluorescence images of dextran uptake by PDAC cells (SW 1990 and MIA PaCa-2) transfected with oe-Ctrl and oe-PGR. (Scale bar, 50 μm) Quantification of macropinocytosis (right panel). *n* = 6 and *p*-value determined by *t*-test. *p ≤ 0.05, **p ≤ 0.01, ***p ≤ 0.001.

Given our findings linking macropinocytosis to PGR expression and activation in PDAC cells, we examined the uptake of TMR-dextran (70 kDa) in Capan-1 and AsPC-1 cells transfected with PGR siRNA or NC siRNA and found that PGR knockdown decreased dextran uptake (Fig. 6A, B). To examine the role of PGR in inducible macropinocytosis in vivo, we assessed the uptake of tumors derived from control Capan-1 cells and PGR-knockdown Capan-1 cells. As expected, PGR knockdown could decrease the TMR-dextran (100 kDa) uptake (Fig. 6D). Furthermore, we validated these findings in PGR overexpressed cell lines (Fig. 6E), yielding similar results.

**Fig. 7:**
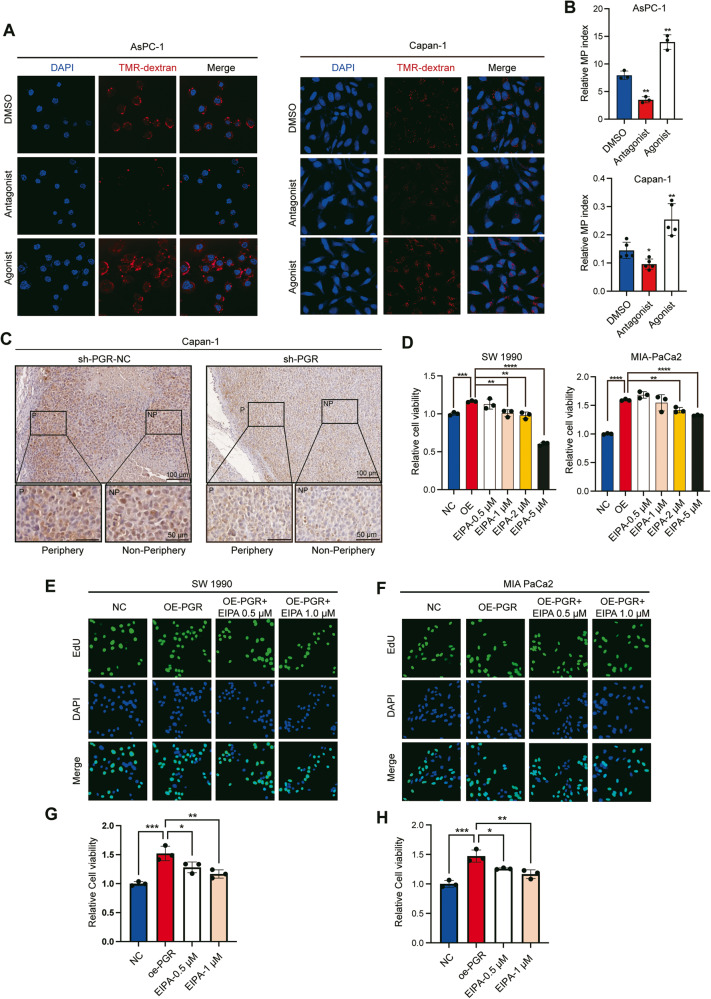
PGR promotes PDAC tumor growth by inducing macropinocytosis. **A** Representative fluorescence images of dextran uptake by PDAC cells (AsPC-1 and Capan-1) treated with agonist (MA) and antagonist (MF) of PGR at a concentration of 10 μM for 24 h. (Scale bar, 50 μm) **B** Quantification of macropinocytosis in **A**. *n* = 6 and *p*-value determined by *t*-test. **C** IHC analyses of the level of PCNA in xenograft pancreatic tumor cells in control and PGR depletion group. Scale bar, 100 μm. **D** CCK8 assay of PDAC cells (SW 1990 and MIA PaCa-2) transfected oe-NC or oe-PGR and treated with different doses of EIPA (0.5, 1.0, 2.0, 5.0 μM). **E**–**H** EdU incorporation assay in PDAC cells (SW 1990 and MIA PaCa-2) transfected oe-NC or oe-PGR and treated with different doses of EIPA (0.5, 1.0 μM). Scale bar, 50 μm. *p ≤ 0.05, **p ≤ 0.01, ***p ≤ 0.001.

The characteristic feature of macropinocytosis is the presence of plasma membrane ruffles that occasionally curve into open, crater-like cups, which are formed by an organized network of actin filaments [7, 27]. We performed immunofluorescence staining with phalloidin to visualize polymerized actin filaments and found that the reduction of PGR led to a decrease in the formation of membrane ruffles in both AsPC-1 and Capan-1 cells (Fig. 6C). To determine whether the observed effects on macropinocytosis were linked to the activity state of PGR, we treated PDAC cells with MA and MF, revealing that the regulation of macropinocytosis was affected by the PGR activity (Fig. 7A, B). Together, these results demonstrate that PGR promotes macropinocytosis in PDAC.

### PGR promotes PDAC growth by inducing macropinocytosis

As an effective way to uptake nutrients, macropinocytosis facilitated the cancer cells in the center of tumors to survive in a more adverse microenvironment. Previous studies revealed that significantly higher levels of macropinocytosis in the non-peripheral areas of tumors relative to the tumor periphery [13]. To understand whether PGR levels might be regulating intratumoral cell proliferation via macropinocytosis, we measured the PCNA staining in PDAC tissue originating from non-peripheral or peripheral regions of xenograft tumor. The results showed a more pronounced downregulation of tumor core proliferation induced by the knockout of PGR (Fig. 7C), suggesting that PGR-induced macropinocytosis might contribute to tumor core growth in an insufficient nutrient environment.

To validate this observation, 5-[N-ethyl-N-isopropyl] amiloride (EIPA), a selective inhibitor of macropinocytosis, was treated in PGR overexpression cultivation. Notably, the beneficial effect of PGR overexpression in PDAC cells (SW 1990, MIA-PaCa2) was suppressed by EIPA (Fig. 7D). The 5-ethynyl-2’-deoxyuridine (EdU) incorporation assay showed that the DNA synthesis rate was raised in PGR-overexpressed cells and suppressed by EIPA (Fig. 7E–H). Altogether, these data demonstrate that the upregulation of PGR in PDAC promotes macropinocytosis to support tumor growth.

### PGR activates macropinocytosis through transcriptional regulation on CDC42 expression

Based on the mRNA array analysis, we observed that PGR knockdown significantly downregulates the expression of CDC42 (Fig. 5E), a regulator of macropinocytosis known to increase macropinocytosis by inducing submembranous actin networks and cytoplasmic membrane ruffling [28, 29]. Consistently, enrichment analysis revealed the potential pathway of the CDC42 GTPase cycle underlying PGR regulation (Fig. 5F). We then analyzed the expression of CDC42 in several GEO data sets (Fig. 5G). Survival analysis implied that the low CDC42 expression level exhibited a survival benefit for PDAC patients (Fig. 5H).

**Fig. 8:**
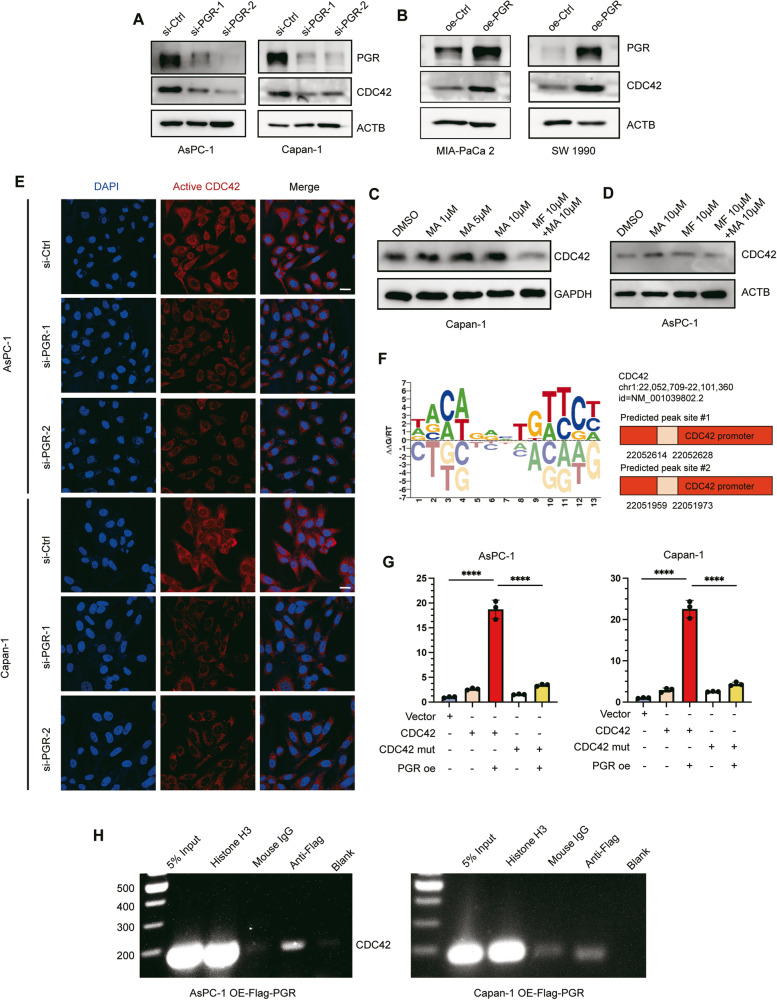
PGR promotes macropinocytosis through regulating CDC42 expression. **A**, **B** Western blotting for PGR and CDC42 in PDAC cells transfected with si-Ctrl, si-PGR, oe-Ctrl and oe-PGR. **C**, **D** Western blotting for CDC42 in PDAC cells (AsPC-1 and Capan-1) treated with different doses of MA (1, 5, 10 μM) and MF (10 μM) for 24 h. **E** Representative immunofluorescence images of PDAC cells transfected with si-Ctrl or si-PGR and labeled with anti-active CDC42 antibody (red) and DAPI (blue). Scale bar, 20 μm. **F** Predicted binding sites of PGR on CDC42 promoter region. **G** Luciferase reporter assays showing the impact of PGR overexpression on CDC42 promoter in PDAC cells (AsPC-1 and Capan-1). **H** ChIP assay to evaluate PGR binding to the promoter region of CDC42 in PDAC cells (AsPC-1 and Capan-1). ****p ≤ 0.0001.

As a nuclear hormone receptor, we hypothesized that PGR regulates CDC42 gene expression by binding to the promoter regions. We detected the expression of CDC42 after both knockdown- and overexpression- PGR, as well as treatment with different doses of MA and MF in PDAC cell cultivation. As expected, the CDC42 expression level was positively related to PGR. PGR activation up-regulated CDC42 level and its inhibition could impair the beneficial effect of MA treatment (Fig. 8A–D). Immunofluorescence staining showed that knockdown of PGR downregulated the activated CDC42 (Fig. 8E). To validate the transcriptional activation, we analyzed the Human Transcription Factor Database (HumanTFDB) and ChIP-seq Database (Cistrome DB) and identified 2 potential binding motifs of PGR (Fig. 8F). By constructing the mutation site of PGR-CDC42 promoter binding site, we utilized luciferase reporters to test whether PGR can drive CDC42 responsiveness. The result showed that the luciferase activity of both CDC42-promoter WT and CDC42-promoter mutant (mut) was comparable, and the luciferase activity dramatically enhanced when co-expressed PGR and CDC42-promoter WT, while no evident difference in CDC42-mut (Fig. 8G). Furthermore, we performed ChIP in PDAC cells overexpressed Flag-PGR and found that PGR can bind to the promoter region of CDC42 (Fig. 8H). In summary, our findings suggest that PGR promotes macropinocytosis by directly regulating CDC42 expression.

## Discussion

Macropinocytosis is a clathrin-independent endocytic pathway, which has emerged as a crucial nutrient supply route in KRAS-transformed PDAC tumors [11]. The pancreatic tumor was notable for dense stroma and a remarkably high mutation rate of KRAS, which was found to be as high as 90% [11]. This dense stroma hinders the exchange of nutrients between tumor cells and blood vessels, resulting in an absolute lack of nutrients within the tumor, particularly with reduced levels of amino acids [30]. Following metabolic remodeling in PDAC cells, amino acid metabolism provides better nutritional support to cells compared to glucose metabolism [31]. In such an environment, small-molecule nutrients from the stroma are insufficient to meet the nutritional needs of rapidly proliferating tumor cells. To address the dilemma, pancreatic cancer cells are able to use macropinocytosis to engulf stromal proteins and degrade them inside the cell in situ [14]. Simultaneously, necessary biological materials can be obtained through the process of macropinocytosis from fragments of dead tumor cells to maintain their own expansion [15]. Studies have shown that enhanced macropinocytosis activity in tumors down-regulates the glucose uptake pathway and the downstream glucose metabolism [32]. Moreover, it has been found that various drug delivery systems can be internalized by KRAS-mutant tumor cells through macropinocytosis, opening up the possibility of using macropinocytosis as an emerging drug delivery method for therapeutic agents [33, 34]. The discovery of macropinocytosis provides new ideas for the study and treatment of pancreatic cancer, emphasizing the urgency of identifying new targets for pancreatic cancer macropinocytosis. This study starts from the perspective of the endocrine system to find key factors regulating macropinocytosis and elucidates that the progesterone receptor can elevate the macropinocytosis level in PDAC cells, thereby maintaining the growth advantage of tumor cells. Our study indicated that macropinocytosis enhanced by PGR down-regulated the availability of glucose and lipids in tumor cells, and this inhibition was able to resume when suppressing macropinocytosis by EIPA (Fig. S2D). As a hinge of macropinocytosis progress, CDC42 has been shown to control the formation of filopodia and cytoskeletal rearrangement independently of Rac [35]. We explored the mechanism underlying PGR-induced macropinocytosis in pancreatic cancer and identified CDC42 as a downstream target of PGR.

Accumulating evidence has shown that metabolism rewiring is a hallmark of cancer [8], which contributes to tumor survival ability and metastatic capacity. Endocrine events are able to regulate tumor cell metabolism including the pentose phosphate pathway [16]. The progesterone receptor (PGR), a steroid-responsive nuclear receptor activated by progesterone, plays pivotal roles in normal mammary gland development, pregnancy, brain function, and cancer advancement [36]. Dysregulated expression of PGR has been associated with cancer initiation and progression, such as hormone-dependent breast cancer, prostate cancer, and colorectal cancer. Elevated PGR expression acts as a tumor suppressor with a significant impact on overall survival [37, 38]. However, the role of progesterone signaling in pancreatic cancer remains elusive. A previous study suggested that overexpression of PGR was linked to favorable overall survival in PDAC patients based on an FGF14 subclassification [39]. Nonetheless, this study only showed the relation between PGR expression and the overall survival in PDAC patients through univariate survival analysis, ignoring the influence of multiple factors, such as tumor stage and differentiation degree. Conversely, in consideration of clinicopathologic characteristics, we performed Kaplan–Meier survival analysis in PDAC patients bearing a tumor diameter smaller than 3 cm and showed that higher PGR expression correlated with poorer prognosis, implying the importance of PGR in tumor initiation. In the present study, We observed that PGR expression exhibited no significant difference between males and females in the TCGA database. The mass spectrometry-based metabolomic profiling of sera from 60 PDAC patients and 40 normal subjects unveiled that the progesterone levels in PDAC patients were nearly 2.5 times higher than those in healthy controls [40]. In investigating the role of PGR in PDAC growth, we generated PGR-knockdown PDAC cells and performed the mRNA array between control and si-PGR PDAC cells. The results we obtained in PDAC were different from those in other cancer types, and in vitro and in vivo functional studies demonstrated that PGR enhances PDAC cell proliferation.

Interestingly, both PGR expression and PGR activation could influence macropinocytosis levels and PDAC growth in our observations (Fig. S1A–C), indicating that the mechanism of PGR functions in pancreatic cancer cells might be greater complex than previously recognized. To pinpoint the potentiate activated factors of PGR without adding supplementary progestin, we detected the content of progesterone in fetal bovine serum, cell-cultured media, and PDAC cell lysates was minimal. Referring to a prior study on non-small cell lung cancer regarding in situ production of progesterone [41], we further observed whether the steroidogenic enzymes (StAR, CYP11A1, HSD3B2) are present and functional in PDAC cells. Our PCR results indicated that PDAC cells possess the capability for autocrine progesterone production (Fig. S1D). Nevertheless, the presence of a non-ligand-independent activation pathway for PGR cannot be ruled out. Previous studies have confirmed that cytoplasmic PKA and SUMO-1 proteins can induce post-translational modifications of PGR, exerting ligand-independent transcriptional activity, including direct DNA binding or affecting the formation of complexes with transcription co-activators [42–44].

Moreover, steroid receptors also mediate extranuclear or rapid actions of cytoplasmic/membrane signaling. The amino-terminal domain of PGR has a specific polyproline-rich (PXPP) motif between aa 421 and 428 that can mediate direct interaction with the SH3 domains of various cytoplasmic signaling molecules, including cSrc, hematopoietic cell kinase (HCK), Fyn, and other kinases or adapter proteins such as PI3K, mTOR and Grb2 [45, 46]. Migliaccio et al. constructed a new PGR mutant that binds ligands but has no transcriptional activity and still stimulates the activation of c-Src and MAP kinases in breast cancer cells, suggesting that the PGR-cScr signaling pathway was functioning independently of transcriptional activation in the nucleus [47]. c-Src kinases that localize to the cytoplasmic side of cellular membranes play a role in membrane trafficking and exhibit four highly conserved SH domains. When c-Src interacts directly with PGR, the former shifts to its active form and promotes the activation of other kinases [48]. c-Src binds to macropinosomes through its N-terminus, including membrane fold formation to centripetal trafficking, and increased kinase activity stimulates macropinosome formation [49, 50]. We propose that PGR has a dual function including directly activating signaling pathways in the cytoplasm through interaction with the SH3 domain of Src kinases or other Ras GTPase proteins to promote macropinocytosis. To test this idea, we performed the immunofluorescence assay of cSrc in PGR control and overexpression PDAC cells. The result revealed that PGR overexpression significantly increased the formation of cSrc-mediated macropinosomes. These results indicated that the interaction between PGR and cSrc could increase its kinase activity and induce the formation of macropinosomes (Fig. S2B). Further interrogation into the downstream molecular mechanisms of PGR leading to regulating macropinocytosis in PDAC, such as non-ligand-dependent pathway or potential interactions with other signaling pathways or factors, is important for future research.

Chemotherapeutic resistance poses a major obstacle to effective tumor therapy. In cancer cells, the inhibition or activation of macropinocytosis could affect the chemotherapeutic resistance, determined by numerous cellular and extracellular factors [51]. Qian et al. demonstrated that inhibiting macropinocytosis in multiple cancer cell lines reduced the internalization of extracellular ATP and its responsibility for drug resistance to tyrosine kinase inhibitors (TKIs) [52]. Another study showed that macropinocytosis offered breast cancer cells amino acids, fatty acids, and nucleotides for biosynthesis by scavenging necrotic cell debris, which confer resistance to therapies targeting tumor anabolism [15]. EIPA inhibits macropinocytosis by inhibiting macropinosome formation in cancer cells with Ras mutations. In human breast cancer cells, different concentrations of EIPA could reverse cisplatin resistance [53], indicating the effectiveness of macropinocytosis inhibition on anticancer drug resistance. However, due to the lack of macropinocytosis-specific targets, it is still hard to cross the barriers to successful therapy. Our work demonstrated that down-regulating the potential gene of macropinocytosis-PGR, boosted the sensitivity of PDAC cells to gemcitabine treatment. Lipid nanoparticle is currently the most widely used drug delivery system, because of its great solubility of small drugs and high pharmacokinetics [54]. Cationic lipid modification of steroids was able to produce remarkable anticancer activity. The recent study synthesized different derivatives of progesterone molecules with self-aggregating properties and defined its selective uptake by cancer cells via macropinocytosis [55]. In future investigation, exploiting the effect of macropinocytosis on anticancer drug delivery and increasing drug sensitivity by the ability of rapid uptake could provide valuable insight into defeating cancer multidrug resistance.

This study introduces groundbreaking insights into the role of PGR as a regulator of macropinocytosis that contributes to nutrient supply in PDAC growth. Our results provide the initial evidence for the participation of PGR in this process, highlighting its potential as a therapeutic target for energy metabolism in pancreatic cancer. Further molecular characterization of PGR will solidify its status as a promising target for macropinocytosis activation, opening up exciting possibilities for the development of new therapies for PDAC.

## Materials and methods

### DNA (cDNA) and mRNA microarray analysis

Total RNA from si-PGR AsPC-1 and Ctrl-PGR AsPC-1 cells were prepared using TRIzol (Invitrogen, USA). The cDNA and mRNA microarray analyses were performed by Shanghai Biotechnology Corporation. The uploaded data is accessed in GSE228301.

### Cell lines, animals, and clinical samples

Human pancreatic cancer cell lines Capan-1, PANC-1, SW 1990, Patu8988, and MIA PaCa-2 were cultured in DMEM-High glucose (BasalMedia, China) media, and AsPC-1, BxPC-3, and CFPAC-1 were maintained in RPMI 1640 Medium (BasalMedia, China). These cell lines were all preserved at Shanghai Cancer Institute, Ren Ji Hospital, School of Medicine, Shanghai Jiao Tong University. The culture medium was supplemented with 1% penicillin-streptomycin (Pen-Strep) (BasalMedia, China) and 10% fetal bovine serum (FBS) (Dcell biologics, China). siRNA duplexes targeting human PGR purchased from GenePharma Company were transfected into cells for 48 h utilizing jetPRIME Polyplus Transfection reagent (Amazon, USA) according to manufacturer’s instructions. The siRNA sequences primer of PGR is listed in Supplementary Table 1. The conditional *LSL-Kras*^*G12D/+*^*; LSL-Trp53*^*R172H/*^*; Pdx1-Cre* (KPC) mice were described previously [2]. BALB/c nude mice used for tumor model establishment were ordered from Shanghai JieSiJie. Animal care and the protocols were carried out by the Guide for the Care and Use of Laboratory Animals prepared by the National Academy of Sciences and published by the NIH (Bethesda, MD). The tumor tissues and non-tumoral adjacent tissues microarray were commercially available. This study was approved by the Ethics Committee of Renji Hospital, Shanghai Jiao Tong University School of Medicine.

### Chromatin immunoprecipitation assay (ChIP-PCR) and PCR analysis

Pancreatic tumor cells (AsPC-1 and Capan-1 cell) transfected with the plasmids of Flag-tagged PGR were prepared for a ChIP assay using a ChIP assay kit (56383 S, Cell Signaling Technology) according to the manufacturer’s protocol. Briefly, PDAC cells were incubated with 1% formaldehyde in the cultured medium for 15 min at room temperature and quenched the reaction using glycine for 5 min. After nuclei preparation and chromatin fragmentation, anti-Flag M2 (2 µg, Sigma-Aldrich, F1804), Histone H3 (2 µg, Cell Signaling, 4620S), or mouse IgG (2 µg, Cell Signaling, 5415S) antibodies were added to prepared IP samples overnight at 4 °C. Immunoprecipitated DNA was purified using a DNA purification kit (Cell Signaling, 14209S). The resulting precipitated DNA samples were analyzed using PCR to amplify a region of the CDC42 promoter with the forward primer 5′- GTGGTTGGGGGAAGGTTGT -3′ and reverse primer 5′- GGAAGCTTCTCTGAAAGGGCTG -3′ (Supplementary Table 2). The PCR products were resolved electrophoretically on a 2% agarose gel and visualized by (Gel image analysis system, Shanghai Furi).

### Luciferase reporter gene assay

Cells were transfected with the indicated CDC42 promoter reporters, siRNAs, or specific gene expression plasmids. The luciferase activity in the cells was quantified using a luciferase assay system 24 h after transfection.

### Ex vivo macropinocytosis assay

For the detection of macropinocytosis in tumors, freshly cut cross-section slices of tumors were subjected to injection (150 μL) and immersion with 10-kDa TMR-dextran (4 mg/mL) at room temperature for 15 minutes. The tissue was rinsed twice in PBS and immediately frozen in optimal cutting temperature (O.C.T.) compound. Tissue processing and image analysis were performed as previously described [56].

### Immunohistochemical and immunofluorescence analysis

For IHC analysis, tissues of xenograft tumors were collected and fixed in 10% formalin followed paraffin embedded and cut into 4-μm sections. Sections from the resected pancreas of KPC mice, xenograft tumors, tissue microarrays, and tumors of human patients with PDAC were conducted and stained for the study purpose. Images were acquired byDigital Slide Scanner (Leica Microsystems, Germany) and analyzed by ImageJ. Engaged antibodies: anti-PGR (8757S, Cell Signaling Technology), anti-PGR (25871-1-AP, Proteintech), and PCNA (GB11010, Servicebio). The IHC score was conducted based on the percentage of positive-stained cells: 0–5% scored 0, 6–35% scored 1, 36–70% scored 2, and more than 70% scored 3. The staining intensity was defined as below: no staining = 0, weakly staining = 1, moderately staining = 2, and strongly staining = 3. The total IHC score was obtained by multiplying the score of intensity and that of percentage score. We defined the total IHC as follows: “-” for a score of 0–1, “+” for a score of 2–3, “++” for a score of 4–6 and “+++” for a score of >6. The samples of “-” and “+” were defined as low expression and the samples of “++” and “+++” were defined as high expression. In the analysis between carcinoma and para-cancerous samples in TMA, a score was assigned to each sample. Samples with a higher score in the carcinoma group were classified as up-regulated, samples with a lower score in the carcinoma group were classified as down-regulated, and samples with the same score were classified as no-change.

For IF analysis of PDAC cells, cells were fixed with 10% (vol/vol) neutral-buffered formalin for 10 min and permeabilized with 0.5% Triton X-100 for 5 min. After blocking with 5% BSA in PBS, the cells were stained with primary antibody, anti-active CDC42 (26905, NewEast Bioscience), overnight at 4 °C and Alexa Fluor 488 secondary antibody (A0423, Beyotime) diluted in 1% BSA for 1 h at room temperature in dark. The cells were then stained with 1 μg/ml DAPI for 30 min to visualize nuclei.

For macropinocytosis detection, cells were seeded on slides and starved in the serum-free medium for 12–18 h. 1 mg/mL TMR-dextran (70 kDa, Thermo Fisher, USA) dissolved in serum-free medium was used for 30 min staining at 37 °C. After incubation, the nuclei were stained by DAPI for 30 min at RT. The samples were examined under Leica confocal microscope (Leica Microsystems, Germany).

### Cell proliferation assay, colony formation

For cell proliferation assay, PDAC cells were plated in 96-well plates at 3000 cells per well and performed using the cell counting kit-8 (CCK-8, Share-Bio, China) according to the manufacturer’s instructions. For detection of the effect of macropinocytosis on cell proliferation, the macropinocytosis inhibitor 5-[N-ethyl-N-isopropyl] amiloride (EIPA) (MCE, China) of 0.5, 1, 2, 5 μM were added respectively in PGR overexpression cells. For colony formation assay, Cells were plated in 6-well plates at 1000 cells per well and colonies were stained with 0.5% crystal violet staining solution after 14 days. The fresh medium was replaced every 3 days.

### Wound healing assay

To assess cell migration, the wound healing assay was performed. Cells at 5 × 10^5^ per well were seeded in six-well plates and treated with plasmid transfection, antagonist, or agonist as figure legend described. After cells grew to 85–95% confluence, we used a 200-μl pipette tip to make a vertical wound and followed three washes with PBS to remove excess cells. Images of the wound were acquired under an inverted microscope (Zeiss AXIOVERT 200) at 0, 24 h.

### Subcutaneous tumor models

BALB/c nude mice (Female, 4-6 weeks of age; Shanghai JieSiJie) were randomly divided in two group and injected subcutaneously with 2 × 10^6^ sh-PGR and wild-type Capan-1 cells at the left flanks. At every 5 days, a subcutaneous intraperitoneal injection of gemcitabine (50 mg/kg) was given to each mouse in the control group and sh-PGR group. After 35 days of post-cell injection, the mice were killed, tumors were excised, and their sizes were measured.

### Western blot analysis

Whole-cell lysates containing equal protein sample loading were used to perform western blot analysis and the protein expression was normalized to β-Actin or GAPDH. The western blot results were quantitated using ImageJ software. The specific antibodies used in western blot were anti-PGR (8757S, Cell Signaling Technology), anti-PGR (25871-1-AP, Proteintech), anti-Beta Actin (81115-1-RR, Proteintech). HRP-conjugated secondary antibodies were obtained from ShareBio (SB-AB0101, SB-AB0102). The agonist and antagonist treatments of PGR were Medroxyprogesterone acetate (M5764, AbMole, USA), and Mifepristone (M3510, AbMole, USA) of 10 μM for 24 h, respectively.

### 5-Ethynyl-2′-Deoxyuridine Incorporation Assay

The 5-ethynyl-2′-deoxyuridine (EdU) incorporation assay was performed using BeyoClick EdU-488 Cell Proliferation Kit (#C0071S, Beyotime), following the manufacturer’s instructions. Cells were seeded on the 8-well chamber and allowed to grow overnight. Then, cells were added medium containing EdU (10 µM) for 2 h incubation, fixed by 4% formaldehyde for 15 min, and permeabilized with 0.3% Triron X-100 in PBS. After PBS wash, cells were incubated with Click reaction cocktail at room temperature for 30 min, followed by nuclei staining with DAPI for 30 min. The images were captured by a Leica Fluorescence Microscope.

### Bioinformatic processing of TCGA and GEO data

Transcriptome data of PDAC patients were obtained from TCGA database and GEO database. After downloading the count matrix, data was normalized using the trimmed mean of M-values (TMM) method. Differentially expressed genes (DEGs) were analyzed using the limma package and identified based on FDR cutoff of 0.05 and a fold change threshold of 2.0. The results were visualized using the ggplot2 package.

### Enrichment analysis

Gene Ontology (GO) and Reactome enrichment analysis of DEGs were performed with the cluster Profiler R package. Terms with a *p*-value < 0.05 were identified as statistically enriched.

### Statistical analyses

Statistical analyses were performed in GraphPad Prism 9.0 and R software. Statistical significance between groups was calculated by Student’s *t*-test or one-way ANOVA. Kaplan–Meier analysis with the log-rank test was used to analyze overall survival. Data were expressed as mean ± SD (ns *p* > 0.05; *p ≤ 0.05, **p ≤ 0.01, ***p ≤ 0.001).

### Supplementary information

Supplementary Figure 1

Supplementary Figure 2

Supplementary Table

Supplementary Legend

## Data Availability

The DNA microarray analysis data generated during the current study have been deposited in the Gene Expression Omnibus (GEO) public database under the accession number GSE228301.
