# Supplementary figures and images for: Progesterone receptor potentiates macropinocytosis through CDC42 in pancreatic ductal adenocarcinoma
Source: Oncogenesis. 2024 Feb 29;13(1):10. doi: 10.1038/s41389-024-00512-7 (PMC10904380; doi:10.1038/s41389-024-00512-7)

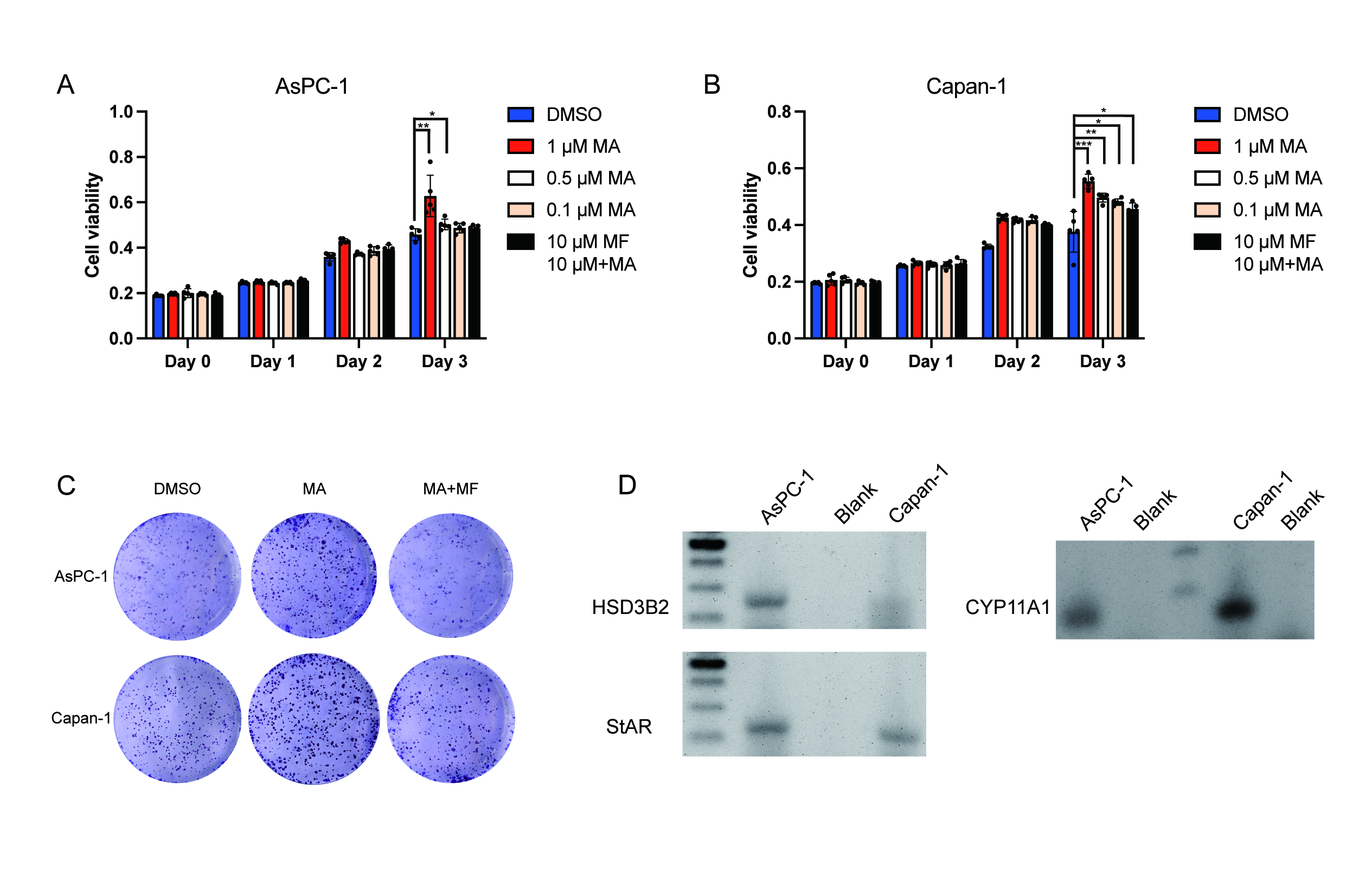

Supplement: Supplementary file 1 — Supplementary Figure 1 [file 41389_2024_512_MOESM1_ESM.tif]

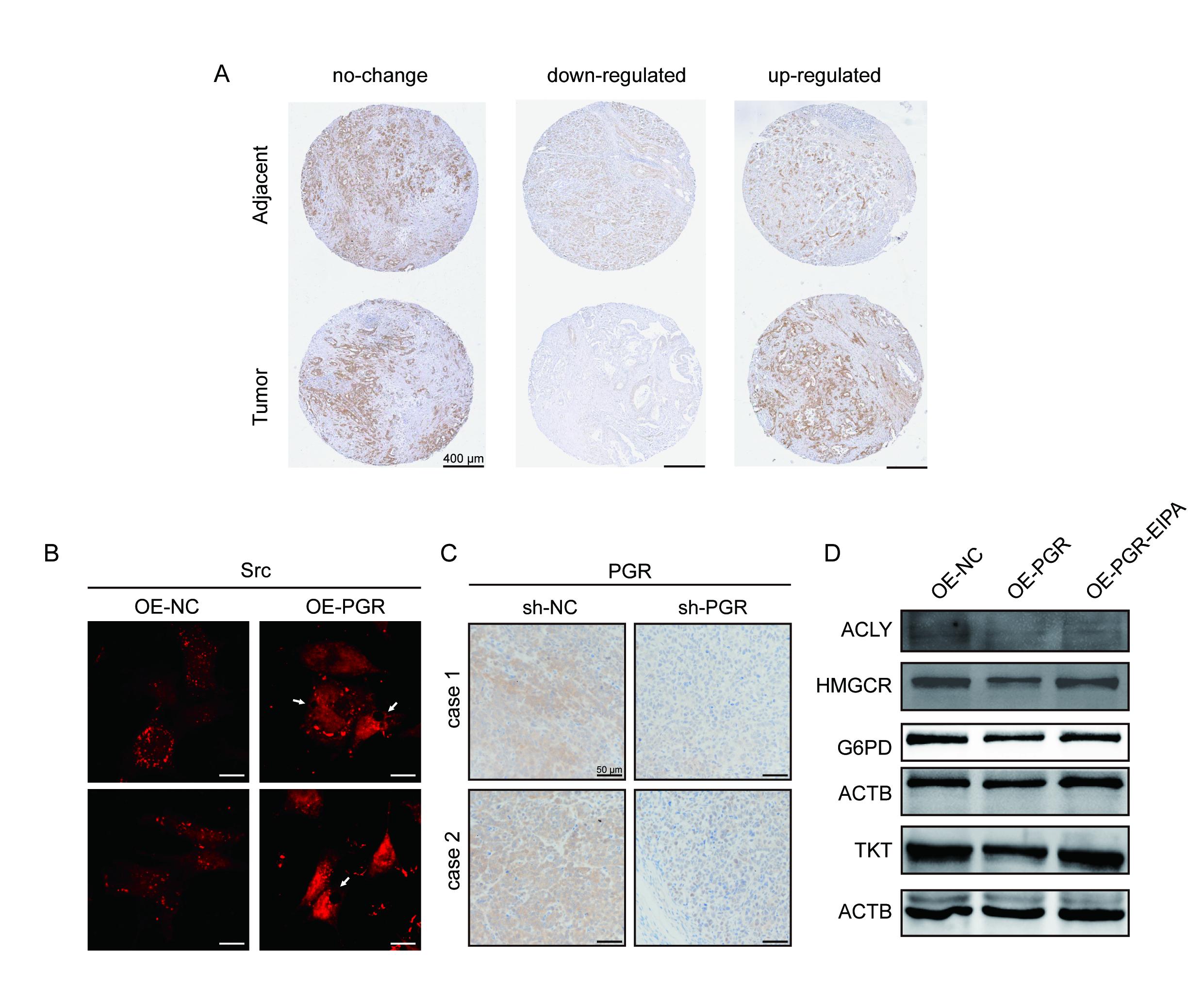

Supplement: Supplementary file 2 — Supplementary Figure 2 [file 41389_2024_512_MOESM2_ESM.tif]
