# Supplementary material for: Progesterone receptor potentiates macropinocytosis through CDC42 in pancreatic ductal adenocarcinoma
Source: Oncogenesis. 2024 Feb 29;13(1):10. doi: 10.1038/s41389-024-00512-7 (PMC10904380; doi:10.1038/s41389-024-00512-7)
Supplement: Supplementary file 3 — Supplementary Table [file 41389_2024_512_MOESM3_ESM.docx]

**Supplementary Table 1: The sequence of siRNA.**

| **siRNA** | **Sequence** |
| --- | --- |
| si-PGR-1 | SS: 5’-GGCAAUUGGUUUGAGGCAATT- 3’  AS: 5’-UUGCCUCAAACCAAUUGCCTT- 3’ |
| si-PGR-2 | SS: 5’-GCUGCACAAUUACCCAAGATT- 3’  AS: 5’-UCUUGGGUAAUUGUGCAGCTT- 3’ |

**Supplementary Table 2: The primer of ChIP-PCR.**

| **Gene** | **Sequence** |
| --- | --- |
| *CDC42* | F: 5’- GTGGTTGGGGGAAGGTTGT - 3’'  R: 5’ - GGAAGCTTCTCTGAAAGGGCTG - 3’ |
