# Supplementary material for: Progesterone receptor potentiates macropinocytosis through CDC42 in pancreatic ductal adenocarcinoma
Source: Oncogenesis. 2024 Feb 29;13(1):10. doi: 10.1038/s41389-024-00512-7 (PMC10904380; doi:10.1038/s41389-024-00512-7)
Supplement: Supplementary file 4 — Supplementary Legend [file 41389_2024_512_MOESM4_ESM.docx]

**Supplementary Figure 1** (A-B) CCK8 assay of PDAC cells (AsPC-1 and Capan-1) treated with different concentration Medroxyprogesterone acetate (MA) and Mifepristone (MF). (C) Colony formation assay of PDAC cells (AsPC-1 and Capan-1) treated with 10 μM Medroxyprogesterone acetate (MA) and Mifepristone (MF). (D) PCR of steroidogenic enzymes (StAR, CYP11A1, HSD3B2) in PDAC cells (AsPC-1 and Capan-1). *p ≤ 0.05, **p ≤ 0.01, ***p ≤ 0.001, ****p ≤ 0.0001.

**Supplementary Figure 2** (A) Representative images of IHC staining for different categorizing cases of PGR in PDAC pancreatic tissue microarray. Scale bar, 400 μm. (B) Representative fluorescence images of Src in PDAC cells transfected with oe-NC or oe-PGR. (Scale bar, 20 μm) (C) Representative images of IHC staining for PGR in subcutaneous tumor tissues. Scale bar, 50 μm. (D) Western blotting for glycolipid metabolism factors in PDAC cells transfected with oe-NC, oe-PGR, and oe-PGR-EIPA (10 μM, 24 h).

**Supplementary Table:** (1) The sequence of siRNA. (2) The primer of ChIP-PCR.
